# Supplementary material for: A fault-intrusion-tolerant system and deadline-aware algorithm for scheduling scientific workflow in the cloud
Source: PeerJ Comput Sci. 2021 Nov 2;7:e747. doi: 10.7717/peerj-cs.747 (PMC8576554; doi:10.7717/peerj-cs.747)
Supplement: Supplemental Information 6 [file peerj-cs-07-747-s006.zip › Myproject1.1/docs/org/cloudbus/cloudsim/network/GraphReaderIF.html]

GraphReaderIF


JavaScript is disabled on your browser.


- Overview
- Package
- Class
- Use
- Tree
- Deprecated
- Index
- Help

- Prev Class
- Next Class

- Frames
- No Frames

- All Classes

- Summary:
- Nested |
- Field |
- Constr |
- Method

- Detail:
- Field |
- Constr |
- Method


org.cloudbus.cloudsim.network

## Interface GraphReaderIF

- All Known Implementing Classes:
  :   GraphReaderBrite

  ---

    

  ```
  public interface GraphReaderIF
  ```

  This interface abstracts an reader for different graph-file-formats

  Since:
  :   CloudSim Toolkit 1.0

- - ### Method Summary

    Methods

    | Modifier and Type | Method and Description |
    | --- | --- |
    | `TopologicalGraph` | `readGraphFile(java.lang.String filename)` this method just reads the file and creates an TopologicalGraph object |

- - ### Method Detail


    - #### readGraphFile

      ```
      TopologicalGraph readGraphFile(java.lang.String filename)
                                     throws java.io.IOException
      ```

      this method just reads the file and creates an TopologicalGraph object

      Parameters:
      :   `filename` - name of the file to read

      Returns:
      :   created TopologicalGraph

      Throws:
      :   `java.io.IOException`


- Overview
- Package
- Class
- Use
- Tree
- Deprecated
- Index
- Help

- Prev Class
- Next Class

- Frames
- No Frames

- All Classes

- Summary:
- Nested |
- Field |
- Constr |
- Method

- Detail:
- Field |
- Constr |
- Method
